# Prognostic role of serum high mobility group box 1 concentration in cardiac surgery

**DOI:** 10.1038/s41598-020-63051-2

**Published:** 2020-04-14

**Authors:** Namo Kim, Sak Lee, Jeong-Rim Lee, Young-Lan Kwak, Ji-Hae Jun, Jae-Kwang Shim

**Affiliations:** 1 Department of Anaesthesiology and Pain Medicine, Yonsei University College of Medicine, Seoul, Republic of Korea; 2 Anaesthesia and Pain Research Institute, Yonsei University College of Medicine, Seoul, Republic of Korea; 3 Department of Thoracic and Cardiovascular Surgery, Yonsei University College of Medicine, Seoul, Republic of Korea

**Keywords:** Biomarkers, Medical research

## Abstract

Outcomes of cardiac surgery are influenced by systemic inflammation. High mobility group box 1 (HMGB1), a pivotal inflammatory mediator, plays a potential role as a prognostic biomarker in cardiovascular disease. The aim of this prospective, observational study was to investigate the relationship between serum HMGB1 concentrations and composite of morbidity endpoints in cardiac surgery. Arterial blood samples for HMGB1 measurement were collected from 250 patients after anaesthetic induction (baseline) and 1 h after weaning from cardiopulmonary bypass (post-CPB). The incidence of composite of morbidity endpoints (death, myocardial infarction, stroke, renal failure and prolonged ventilator care) was compared in relation to the tertile distribution of serum HMGB1 concentrations. The incidence of composite of morbidity endpoints was significantly different with respect to the tertile distribution of post-CPB HMGB1 concentrations (p = 0.005) only, and not to the baseline. Multivariable analysis revealed post-CPB HMGB1 concentration (OR, 1.072; p = 0.044), pre-operative creatinine and duration of CPB as independent risk factors of adverse outcome. Accounting for its prominent role in mediating sterile inflammation and its relation to detrimental outcome, HMGB1 measured 1 h after weaning from CPB would serve as a useful biomarker for accurate risk stratification in cardiac surgical patients and may guide tailored anti-inflammatory therapy.

## Introduction

Despite advances in perioperative care, cardiac surgery continues to be associated with an increased risk of unfavourable outcomes^1^. In conjunction with patient-related factors, systemic inflammation has been recognized as a key factor for post-operative complications, including multi-organ failure or even death^2^. Nevertheless, studies addressing the prognostic role of non-specific serum markers of inflammation in cardiac surgery yielded conflicting results^3–5^, not to mention that indiscriminate anti-inflammatory therapies represented by using steroids did not prove to be beneficial^6^.

High-mobility group box 1 (HMGB1) is an important mediator of sterile inflammatory responses in organ damage; it is either released from the nucleus of necrotic cells or actively secreted from inflammatory cells^7^. Accordingly, studies have elucidated the role of increased serum HMGB1 concentration in acute coronary syndrome, atherosclerosis, heart failure, and other cardiovascular diseases as a marker for inflammatory response and detrimental outcome^8–11^. However, no studies have examined the prognostic role of serum HMGB1 levels in cardiac surgery, while the importance of a reliable biomarker reflecting extensive systemic inflammation cannot be overemphasized in terms of accurate risk stratification and as a potential guide for selective application of therapies in that regard. This prospective, observational study aims to investigate the prognostic role of HMGB1 through identifying the relationship between serum HMGB1 concentrations and the incidence of the composite of morbidity endpoints in patients undergoing complex cardiac surgery.

## Materials and Methods

### Study population

The study protocol was approved by the Institutional Review Board and Hospital Research Ethics Committee of Severance Hospital at Yonsei University College of Medicine (Protocol No. 4-2014-0764) and was registered at clinicaltrials.gov (NCT02490644; date of registration, January 12, 2015). All study methods were performed in accordance with the relevant guidelines and regulations and written informed consent was obtained from all patients. The trial was conducted at Severance Cardiovascular Hospital, Yonsei University College of Medicine, Seoul, Korea between January 2015 and August 2017.

A total of 253 patients scheduled for elective valvular heart surgery were consecutively enrolled. Inclusion criteria were patients who received aortic valve replacement, valve replacement that involved more than single valve, valve replacement with coronary artery bypass graft or aortic graft replacement surgery. Exclusion criteria were patients with acute coronary syndrome, infectious diseases including endocarditis, malignancies, emergency operations and patients who had previous or intra-operative exposure to steroids.

### Perioperative management

Perioperative management, including cardiopulmonary bypass (CPB) and intensive care unit (ICU) care, was performed according to institutional standardized protocols. In brief, anaesthetic monitoring included a pulmonary artery catheter and transoesophageal echocardiography. Anaesthesia was induced with midazolam (0.03–0.05 mg/kg) and sufentanil (1.0–2.0 μg/kg) and was maintained with sevoflurane and continuous infusion of sufentanil (0.5–1.0 μg/kg/h) guided by the bispectral index score. CPB was operated with nonpulsatile perfusion at pump flow rates of 2.2–2.5 l/min/m^2^ using alpha-stat pH and tepid body temperature management (cooled to 32–34 °C). During surgery, a cell salvage device and tranexamic acid (loading dose: 1 g, additional 1 g mixed to CPB prime followed by an infusion of 200 mg/h during surgery) were used in all patients.

Target mean arterial pressure throughout the perioperative period was 60–80 mmHg. First-line vasopressor was norepinephrine (up to 0.5 μg/kg/min) with the addition of vasopressin (up to 4 IU/h) in a stepwise manner. First-line inotropic agent was milrinone with addition of dobutamine and/or epinephrine in a stepwise manner, as necessary. First-line vasodilator was nicardipine or isosorbide dinitrate depending on the patient’s condition with the aid of nitroprusside for acute control, as necessary. Transfusion trigger for packed erythrocytes (pRBC) was 8 g/dL.

### Outcome measurement

Arterial blood samples were collected twice at the following time points; after anaesthetic induction (baseline) and 1 h after weaning from CPB (post-CPB). Blood samples were immediately centrifuged and stored at −80 °C for subsequent HMGB1 measurement, which was done by enzyme-linked immunosorbent assay (HMGB1 ELISA kit, REF. ST51011, Standard range = 0 ~ 20 ng/mL, IBL, Hamburg, Germany).

The primary outcome was to evaluate and compare the incidence of composite of morbidity endpoints in relation to the tertile distribution of HMGB1 concentrations. The composite of morbidity endpoints was modified from the Society of Thoracic Surgeons cardiac surgery risk models^12^ and defined as follows: ➀ In-hospital death from any cause; ➁ permanent disability caused by stroke: a central neurologic deficit persisting longer than 72 hours; ➂ post-operative myocardial infarction (MI): increase in troponin-T 0.5 ng/ml (5 times the upper normal limit) and/or development of new pathological Q-waves on the electrocardiogram; ➃ renal failure: a new requirement for dialysis or an increase of the serum creatinine to greater than 2.0 mg/dL and double the most recent pre-operative creatinine level; and ➄ prolonged ventilator care >24 h. The secondary endpoint was to evaluate risk factors for the incidence of composite of morbidity endpoints including serum HMGB1 concentrations.

Assessed pre-operative variables included demographic data, hypertension, diabetes mellitus, cerebrovascular accident, coronary artery disease including previous MI, congestive heart failure (New York Heart Association functional classification III or IV), left ventricular ejection fraction, pre-operative creatinine, medication use, and EuroSCORE II. Intra-operative variables included the type of cardiac procedures, type of valves, and duration of CPB. Postoperative variables included serum creatinine, blood loss, transfusion and the number of days of ICU care and post-operative hospitalization.

### Statistical analysis

The sample size was calculated based on institutional data of patients undergoing valvular heart surgery in which the overall incidence of composite of morbidity endpoints was approximately 30%. We determined that 81 patients would be required for each tertile to detect a 2-fold increase in the incidence of composite of morbidity endpoints in the third tertile compared to 20% incidence of the first tertile in relation to HMGB1 concentrations at an alpha level of 0.05 with 80% power.

The measured baseline and post-CPB concentration were compared with Wilcoxon signed-rank test. Continuous variables among the tertiles were compared by one-way ANOVA with Bonferroni post-hoc test for normally distributed values; otherwise, the Kruskal-Wallis test was used. Proportions were compared by Fisher’s exact test or chi-squared test as appropriate. The ability of HMGB1 concentrations and other variables to predict the composite of morbidity endpoints were evaluated by receiver operator characteristic curve analysis. The optimal cut-off value for HMGB1 concentrations was defined as the point on the receiver operator characteristic curve providing the greatest sum of sensitivity and specificity.

The odds ratios and 95% confidence intervals (CIs) investigating the independent predictive role of HMGB1 to the composite of morbidity endpoints were assessed by logistic regression. After the univariable analysis, parameters with p < 0.05 were enlisted to the final model of multivariable logistic regression analysis.

Categorical variables are expressed as number (%) and continuous variables are expressed as mean ± standard deviation or median [interquartile range] as appropriate, with p < 0.05 considered statistically significant. Statistical analyses were performed using SPSS version 23.0 (SPSS Inc., Chicago, IL, USA).

## Results

**Figure 1:**
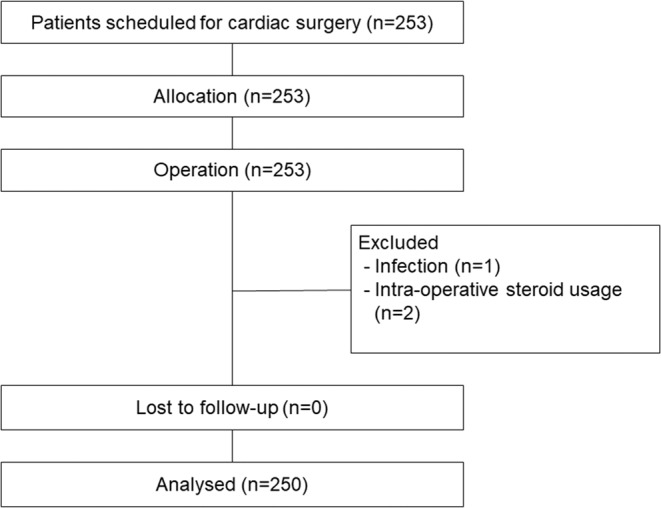
Flowchart of patient enrolment.

Among the 253 patients who were screened for the study, 3 were excluded (Fig. 1). The median baseline and post-CPB HMGB1 concentration were 0.620 [0.268–1.551] ng/ml and 3.933 [2.018–6.866] ng/ml, respectively. Post-CPB HMGB1 concentration was increased in 211 patients compared to baseline HMGB1 concentration.

**Figure 2:**
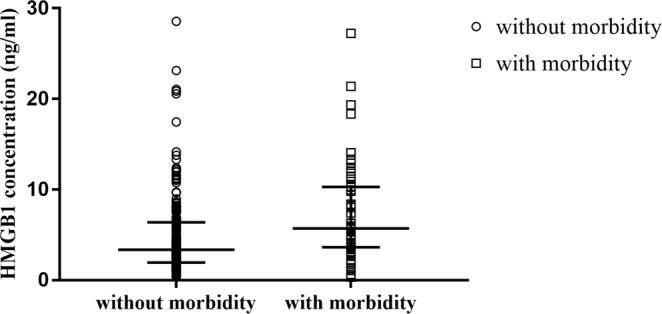
Scatter plot of post-CPB HMGB1 concentrations between patients who exhibited composite of morbidity endpoints or not. The horizontal lines shown in the graph represent median [interquartile range] of each group, which were 3.370 [1.935–6.385] and 5.703 [3.627–10.282], respectively (p < 0.001). HMGB1 = High mobility group box 1.

There was a significant difference in the incidence of composite of morbidity endpoints in relation to the tertile distribution of HMGB1 concentrations measured post-CPB (p = 0.005, Table 1), but not in relation to baseline HMGB1 concentrations measured after anaesthetic induction (p = 0.537, Table 2). Scatter plot of the post-CPB HMGB1 concentrations between patients who exhibited composite of morbidity endpoints or not also demonstrated a significant difference (Fig. 2). Accordingly, further analyses were performed according to the tertile distribution of post-CPB HMGB1 concentrations.

**Table 1:** Composite of morbidity endpoints in relation to tertile distribution of the post-CPB HMGB1 concentration.

	Total(n = 250)	First Tertile (n = 83)(0.337–2.684 ng/ml)	Second Tertile (n = 83)(2.689–5.770 ng/ml)	Third tertile (n = 84)(5.799–28.552 ng/ml)	P value
HMGB1, ng/ml	3.933 [2.018–6.866]	1.734 [1.088–2.019]	3.922 [3.209–4.500]*	8.443 [6.829–12.029]*^†^	<0.001
Composite morbidity, n (%)	58 (23)	10 (12)	20 (24)*	28 (33)*	0.005
Death, n (%)	10 (4)	0 (0)	4 (5)	6 (7)	0.039
Stroke, n (%)	4 (2)	2 (2)	2 (2)	0 (0)	0.401
Myocardial infarction, n (%)	0 (0)				—
Renal failure, n (%)	29 (12)	2 (2)	9 (11)	18 (21)*	<0.001
Prolonged ventilation> 24 h					
Number, n (%)	33 (13)	7 (8)	11 (13)	15 (18)	0.205
Duration (hrs)	17 [14–21]	18 [13–21]	17 [13–20]	17 [15–22]	0.786

Values are expressed as the numbers of patients (%), median [interquartile range]. *P < 0.05 vs. first tertile; ^†^P < 0.05 vs. second tertile.

CPB = cardiopulmonary bypass; HMGB1 = high mobility group box 1.

**Table 2:** Composite of morbidity in relation to tertile distribution of the baseline HMGB1 concentration.

	Total(n = 250)	First Tertile (n = 83)(0.001–0.348 ng/ml)	Second Tertile (n = 83)(0.351–0.967 ng/ml)	Third tertile (n = 84)(0.982–47.799 ng/ml)	P value
HMGB1, ng/ml	0.620 [0.268–1.551]	0.187 [0.095–0.269]	0.620 [0.483–0.816]*	3.144 [1.520–7.584]*^†^	<0.001
Composite morbidity, n (%)	58 (23)	16 (19)	22 (27)	20 (24)	0.537
Death, n (%)	10 (4)	4 (5)	4 (5)	2 (2)	0.669
Stroke, n (%)	4 (2)	3 (4)	1 (1)	0	0.131
Myocardial infarction, n (%)	0 (0)				—
Renal failure, n (%)	29 (12)	9 (11)	13 (16)	7 (8)	0.338
Prolonged ventilation> 24 h					
Number, n (%)	33 (13)	6 (7)	11 (13)	16 (19)	0.082
Duration (hrs)	17 [14–21]	17 [12–20]	18 [15–22]	18 [15–22]	0.251

Values are expressed as the numbers of patients (%), median [interquartile range]. *P < 0.05 vs. first tertile; ^†^P < 0.05 vs. second tertile.

HMGB1 = High mobility group box 1.

The characteristics of the patients at baseline were comparable among the tertiles except for sex, congestive heart failure, and EuroSCORE II (Table 3). There were significant differences in the duration of CPB, length of ICU stay, and length of hospital stay in relation to the tertile distribution of the post-CPB HMGB1 concentrations (Table 4).

**Table 3:** Patient characteristics in relation to tertile distribution of post-CPB HMGB1 concentration.

	Total(n = 250)	First tertile (n = 83)(0.337–2.684 ng/ml)	Second tertile (n = 83)(2.689–5.770 ng/ml)	Third tertile (n = 84)(5.799–28.552 ng/ml)	P value
Age, yrs	66.1 ± 11.3	66.0 ± 10.2	67.0 ± 11.8	65.1 ± 11.9	0.556
Gender (M/F)	119/131	53/30	41/42	25/59	<0.001
Body mass index, kg/m^2^	23.5 ± 5.7	24.2 ± 7.3	24.0 ± 4.4	22.7 ± 5.0	0.233
Hypertension, n (%)	140 (56)	50 (60)	49 (59)	41(49)	0.263
Diabetes mellitus, n (%)	59 (24)	22 (27)	23 (28)	14 (17)	0.187
Cerebrovascular accident, n (%)	21 (8)	9 (11)	9 (11)	3 (4)	0.128
Coronary artery disease, n (%)	60 (24)	15 (18)	27 (33)	18 (21)	0.073
Single-vessel	26 (10)	11 (13)	10 (12)	5 (6)	
Double-vessel	17 (7)	4 (5)	7 (8)	6 (7)	
Triple-vessel	17 (7)	0 (0)	10 (12)	7 (8)	
Previous myocardial infarction, n (%)	14 (6)	3 (4)	8 (8)	3 (4)	0.175
Congestive heart failure (NYHA III,IV), n (%)	73 (29)	13 (16)	29 (35)	31 (37)	0.004
Left ventricular ejection fraction, n (%)	60 ± 13	62 ± 13	58 ± 14	60 ± 11	0.175
Preoperative creatinine, mg/dl	0.88 [0.68–1.11]	0.86 [0.67–0.99]	0.87 [0.73–1.27]	0.90 [0.67–1.13]	0.272
Preoperative medication, n (%)					
Beta-blockers	85 (34)	26 (31)	32 (39)	27 (32)	0.574
Renin-angiotensin system antagonists	110 (44)	29 (35)	39 (47)	42 (50)	0.118
Calcium-channel blockers	74 (30)	23 (28)	27 (33)	24 (29)	0.802
EuroSCORE II	6.2 ± 3.3	5.1 ± 3.3	6.4 ± 3.3*	6.9 ± 3.1*	0.001

Values are expressed as the numbers of patients (%), mean ± standard deviation, median [interquartile range]. *P < 0.05 vs. first tertile.

CPB = cardiopulmonary bypass; HMGB1 = High mobility group box 1; NYHA = New York Heart Association.

**Table 4:** Perioperative data in relation to tertile distribution of post-CPB HMGB1 concentration.

	Total(n = 250)	First tertile (n = 83)(0.337–2.684 ng/ml)	Second tertile (n = 83)(2.689–5.770 ng/ml)	Third tertile (n = 84)(5.799–28.552 ng/ml)	P value
Cardiac procedure, n (%)					0.076
Valve	213 (85)	76 (91)	63 (76)	74 (88)	
Valve and CABG	22 (9)	4 (5)	12 (14)	6 (7)	
Valve and aortic procedure	15 (6)	3 (4)	8 (10)	4 (5)	
Type of valve, n (%)					0.095
Mechanical	93 (37)	26 (31)	28 (34)	39 (46)	
Bioprosthesis	157 (63)	57 (69)	55 (66)	45 (54)	
Cardiopulmonary bypass, min	115 [85–151]	98 [70–117]	115 [85–150]*	149 [103–200]*^†^	<0.001
Postoperative blood loss, ml	704 [502–763]	715 [500–760]	600 [500–760]	730 [512–900]	0.123
Postoperative pRBC transfusion, n %	56 (22)	22 (27)	18 (22)	16 (19)	0.508
Intensive care unit stay, day	3 [2–4]	2 [2,3]	3 [2,3]	3 [2–5]	0.030
Hospital stay, day	15 [11–18]	12 [9–15]	15 [11–18]*	18 [13–29]*^†^	<0.001

Values are expressed as the numbers of patients (%), median [interquartile range]. *P < 0.05 vs. first tertile; ^†^P < 0.05 vs. second tertile. CPB = cardiopulmonary bypass; HMGB1 = High mobility group box 1; CABG = coronary artery bypass graft; pRBC = packed erythrocytes.

The incidence of composite of morbidity endpoints was significantly different among the tertiles (1^st^ tertile: 12%, 2^nd^ tertile: 24%, 3^rd^ tertile: 33%, p = 0.005). Among the specific morbidity endpoints, death and the incidence of renal failure were significantly different in relation to the tertile distribution of post-CPB HMGB1 concentrations (Table 1).

In univariable analysis for identifying predictors of composite of morbidity endpoints, post-CPB HMGB1 concentrations, coronary artery disease, congestive heart failure, EuroSCORE II, pre-operative creatinine, and duration of CPB exhibited p < 0.05. In multivariable analysis of these variables, post-CPB HMGB1 concentrations, pre-operative creatinine, and duration of CPB remained as independent predictors (Table 5). The Pearson β correlation coefficient of post-CPB HMGB1 concentrations and CPB duration was 0.373 (p < 0.001).

**Table 5:** Predictive power of selected variables for the composite of morbidity endpoints.

Predictors	Univariable Analysis			Multivariable Analysis		
	Odds ratio	95% CI	P value	Odds ratio	95% CI	P value
HMGB1	1.098	1.036–1.164	0.002	1.072	1.002–1.148	0.044
Coronary artery disease	2.753	1.456–5.204	0.002	1.807	0.838–3.899	0.131
Congestive heart failure	2.060	1.114–3.811	0.021	1.390	0.648–2.979	0.398
EuroSCORE II	1.155	1.057–1.262	0.001	1.023	0.912–1.148	0.694
Pre-operative creatinine	2.349	1.494–3.691	<0.001	2.107	1.302–3.409	0.002
Cardiopulmonary bypass time	1.011	1.005–1.017	<0.001	1.010	1.002–1.017	0.009

CI = confidence interval, HMGB1 = high mobility group box 1.

In the receiver operator characteristic curve analysis, the optimal cut-off concentration of post-CPB HMGB1 for predicting the composite of morbidity endpoints was 4.474 ng/ml (area under the receiver operator characteristic curve [AUROC], 0.664; 95% confidence interval, 0.584–0.743; p < 0.001), which yielded an odds ratio of 2.476 (95% confidence interval; 1.156–5.303, p = 0.020) in the multivariable analysis adjusting for other confounders. The optimal cut-off concentrations for death and renal failure were 4.905 ng/ml (AUROC, 0.726; 95% confidence interval, 0.619–0.833; p = 0.016) and 4.905 ng/ml (AUROC, 0.717; 95% confidence interval, 0.627–0.807; p < 0.001), respectively. The optimal cut-off of CPB duration for adverse outcome was 125 min (AUROC, 0.659; 95% confidence interval, 0.581–0.738; p < 0.001) yielding an odds ratio of 3.209 (95% confidence interval; 1.530–6.730, p = 0.002) when introduced to the multivariable analysis.

## Discussion

This prospective, observational study demonstrates that patients with high serum HMGB1 concentrations 1 h after weaning from CPB were associated with a significantly increased risk of developing composite of morbidity endpoints after cardiac surgery, renal failure and death in particular. In addition, HMGB1 concentration was shown to be an independent predictor of adverse outcomes, along with pre-operative creatinine and CPB duration.

In cardiac surgery, sterile inflammatory response is caused by CPB, myocardial damage with ischemia/reperfusion injury, and surgical stimulation^13^. While fundamentally being a protective internal response, it progresses to a systemic inflammatory response syndrome when extensive, which is associated with major complications such as multiple organ damage and even mortality after surgery^14–16^. Thus, identifying patients at risk of aggravated systemic inflammation through a reliable serum biomarker would be of clinical importance in terms of accurate risk stratification and guiding tailored anti-inflammatory therapies.

HMGB1 is present in the nucleus of almost all cells, functioning as a DNA chaperone^17^. Over the past decade, the dominant role of HMGB1 as an early and late mediator of inflammation has been well-recognized, where it migrates from the nucleus into the cytoplasm or is secreted outside the cell by various disease processes^7,18,19^. Well-known downstream molecular pathways that further aggravate systemic inflammation and tissue injury involve the activation of toll-like receptors, receptors for advanced glycation products, and NF-κB^20^. Accordingly, increases in the concentration of HMGB1 in the blood has been implicated as a potential biochemical marker of inflammation linked to outcomes in clinical practice^21^. Indeed, the concentration-dependent activity of HMGB1 has been shown to be associated with the occurrence, development, and prognosis of various cardiovascular diseases by recent studies that include acute coronary syndrome, MI, and heart failure^11,22,23^. Despite its potential prognostic importance in cardiovascular disease, its role as a predictor of outcome in cardiac surgery has not been tested heretofore.

In the current trial, our results revealed that patients in the third tertile group with higher levels of post-CPB HMGB1 concentrations reflecting extensive systemic inflammation had a significantly greater incidence of composite of morbidity endpoints and a significantly longer duration of post-operative hospitalization. Moreover, multivariable logistic regression analysis adjusting for possible confounders revealed post-CPB HMGB1 concentrations as an independent predictor of adverse outcomes with a cut-off value of 4.474 ng/ml yielding a 2.5-fold increased risk of composite of morbidity endpoints. In detail, among the assessed composite of morbidity endpoints, HMGB1 concentrations showed statistical significance for predicting the mortality and renal failure. The corresponding optimal cut-off value of HMGB1 concentration was 4.905 ng/ml in the current study, which is similar to those reported in ST-elevation myocardial infarction or heart failure patients^10,11^.

Notably, post-induction HMGB1 concentrations were not linked to adverse outcomes, indicating that the baseline inflammatory status of the patients does not necessarily lead to extensive systemic inflammatory response after surgery. This contrasts with the prognostic value of pre-operative (baseline) high sensitivity C-reactive protein (hsCRP) in patients undergoing coronary artery bypass surgery, which has been shown to be related to an increased risk of major cardiovascular and cerebral events^24^. However, taking into account its characteristics as a nonspecific acute phase reactant, hsCRP seems to be more closely related to the vascular risks such as plaque rupture and vascular thrombosis, showing its predictive role to be limited to the cardiovascular events in patients with coronary and/or metabolic disease^5^.

Congruent to our results, CPB duration^25^ and pre-operative creatinine^26^ are well-recognized risk factors of post-operative morbidity and mortality in cardiac surgery. A prolonged duration of CPB may affect the level of post-CPB HMGB1. However, the correlation between post-CPB HMGB1 concentrations and CPB duration was weak (β coefficient = 0.373), while the independence of these variables on predicting outcome could be verified in multivariable logistic regression analysis. Therefore, our results implicate that extensive and harmful inflammatory response cannot be anticipated simply by the patient’s baseline inflammatory status or the duration of CPB adding value to the prognostic importance of post-CPB HMGB1 concentrations in that regard. Of note, inflammatory response is meant to be protective, which is possibly the main reason why studies validating the efficacies of indiscriminate anti-inflammatory measures on outcomes after cardiac surgery yielded conflicting results^6^. Whether tailored anti-inflammatory therapy guided by post-CPB HMGB1 concentrations would actually improve outcome remains to be proven by future studies.

The current study is subject to the following limitation. First, although we assessed HMGB1 concentrations 1 h after weaning from CPB according to a previous study that demonstrated a peak increase in HMGB1 at that time point with significant correlation to the increase of inflammatory cytokines^27^, it remains obscure whether HMGB1 concentrations measured at other time points would yield different prognostic values. Second, since our study involved complex valvular heart surgeries, caution should be exercised not to extrapolate our results to other cardiac surgeries. Third, although we have excluded conditions that may confound HMGB1 secretion such as malignancy and infection, HMGB1 may be secreted by various disease conditions such as autoimmune diseases that may not have been fully controlled in our study. Finally, concomitant assessments of other well-validated serum inflammatory indicators would have provided more insights on the role of HMGB1.

In conclusion, the current study provides primary evidence that HMGB1 concentration measured 1 h after weaning from CPB is independently associated with adverse outcomes after cardiac surgery. Systemic inflammatory response cannot be held solely responsible for poor prognosis after cardiac surgery. Yet, considering its important role in conveying major organ injury, post-CPB serum HMGB1 concentration may serve as a useful biomarker for accurate risk stratification in cardiac surgical patients.
